# Decreased prefrontal glutamatergic function is associated with a reduced astrocyte-related gene expression in treatment-resistant depression

**DOI:** 10.1038/s41398-024-03186-2

**Published:** 2024-11-25

**Authors:** Masataka Wada, Shinichiro Nakajima, Shiori Honda, Mayuko Takano, Keita Taniguchi, Saki Homma, Risako Ueda, Yui Tobari, Yu Mimura, Shinya Fujii, Masaru Mimura, Yoshihiro Noda

**Affiliations:** 1 Department of Neuropsychiatry, Keio University School of Medicine, Tokyo, Japan; 2 Department of Psychiatry and Behavioral Sciences, Stanford University, Stanford, CA USA; 3 Teijin Pharma Ltd., Tokyo, Japan; 4 Faculty of Environment and Information Studies, Keio University, Tokyo, Japan; 5 Department of Psychiatry, International University of Health and Welfare, Mita Hospital, Tokyo, Japan

**Keywords:** Physiology, Diagnostic markers, Depression, Pathogenesis, Neuroscience

## Abstract

Glutamatergic dysfunction is involved in the pathophysiology of treatment-resistant depression (TRD). However, few physiological studies have evaluated its pathophysiology in vivo in individuals with TRD. Transcranial magnetic stimulation-electroencephalography (TMS-EEG) techniques can assess intracortical facilitation (ICF), which reflects glutamatergic neurophysiological function in specific cortical regions. The objectives of this study were (1) to compare glutamatergic receptor-mediated function as indexed with ICF TMS-EEG in the dorsolateral prefrontal cortex (DLPFC) between participants with TRD and healthy controls (HCs) and (2) to explore the relationships between cell-specific gene expression levels and the group difference in glutamatergic neural propagation using virtual histology approach. Sixty participants with TRD and thirty HCs were examined with ICF TMS-EEG measure (80 single-pulse TMS and paired-pulse ICF) in the left DLPFC. Both sensor and source-level ICF measures were computed to compare them between the TRD and HC groups. Furthermore, we conducted spatial correlation analyses interregionally between ICF glutamatergic activity and cell-specific gene expression levels employing the Allen Human Brain Atlas dataset. DLPFC-ICF at the sensor level was not significantly different between the two groups, whereas DLPFC-ICF at the source level was reduced in the TRD group compared with the HC group (*p* = 0.026). Moreover, the reduced ICF signal propagation of TRD correlated with astrocyte-specific gene expression level (*p* < 0.0001). The glutamatergic neural activities indexed by ICF in the left DLPFC were decreased in participants with TRD. Additionally, a relative reduction in glutamatergic signal propagation originating from the DLPFC in TRD may be associated with astrocytic abnormality.

## Introduction

Major depressive disorder (MDD) is one of the most widespread mental illnesses [1]. However, approximately one-third of individuals with MDD do not respond to conventional antidepressants, which is called treatment-resistant depression (TRD) [2]. Individuals with TRD have a significantly reduced quality of life, exhibit marked activity impairment, and require greater medical resources compared with individuals with non-TRD [3]. Thus, elucidating the pathophysiology of TRD and developing effective treatments based on the neural basis are urgently needed.

A host of research, including genetic, postmortem, and clinical studies, has demonstrated that impaired glutamatergic neural function is the pathophysiological basis of MDD [4–7]. The glutamate hypothesis of depression is corroborated by the fact that ketamine, a glutamatergic *N*-methyl-d-aspartate (NMDA) receptor antagonist, has an odds ratio of 6.33 (95% CI: 3.33 to 12.05) for the response rate in individuals with TRD [8]. To date, however, there is the only study that employed proton magnetic resonance spectroscopy (^1^H-MRS), reporting decreased levels of glutamate + glutamine in the anterior cingulate cortex of individuals with TRD compared with healthy controls (HCs), while no difference was found between the non-TRD and HC groups [9]. Hence, further research is needed to elucidate the glutamatergic neural dysfunction of TRD.

Neuroimaging studies on depression have consistently identified the dorsolateral prefrontal cortex (DLPFC) as a critical hub region in MDD [10–15]. Specifically, Padmanabhan et al. performed a lesion network mapping study using data from 400 post-lesion depressed individuals and revealed that the left DLPFC was a central hub within the brain circuit, functionally connected to the lesion sites associated with depression [16]. Additionally, Siddiqi et al. demonstrated that lesions associated with depressive symptoms overlapped with brain regions and circuits that are modulated by transcranial magnetic stimulation (TMS) and deep brain stimulation for individuals with TRD [17]. Remarkably, the DLPFC was one of the common sites that displayed robust connectivity to the lesion sites or targeted regions in all three different modalities (i.e., lesion mapping, TMS, and deep brain stimulation) [17]. These findings suggest that dysfunction of the DLPFC represents a common and characteristic neural basis for depressive symptoms, even in a highly heterogeneous TRD population.

Intracortical facilitation (ICF) paradigm can assess primarily NMDA receptor-mediated neural activity, in the cortical region of interest by applying the combined TMS-electroencephalography (EEG) method [18, 19]. This method was established by the facilitation of motor-evoked potential in the electromyography (EMG) responses with paired-pulse TMS (first stimulus at subthreshold and second stimulus at suprathreshold) with an interstimulus interval of 10 ms, compared to single-pulse TMS (test stimulus at suprathreshold) to the motor cortex [20]. Of note, a meta-analysis reported that patients with MDD have increased ICF in their M1 region, suggesting enhanced glutamatergic function in M1 among patients with MDD [21]. The validity of the ICF paradigm has been demonstrated by the suppression of ICF with the administration of glutamate NMDA receptor antagonists, establishing ICF as an indicator of glutamatergic neurotransmission through NMDA receptors [18, 19]. Recently, TMS-EEG has been applied in conjunction with TMS-EMG to evaluate glutamate receptor-mediated activity in various cortical regions, including the DLPFC, beyond the motor cortex [21–23]. As the conventional TMS-EEG method has been limited by the potential peripheral nerve stimulation-elicited artifacts, we employed a sophisticated approach based on EEG signal source estimation [24]. To date, no study has investigated the glutamatergic function indexed by ICF TMS-EEG in individuals with TRD.

In this context, the present study primarily focused on the examination of glutamatergic dysfunction in individuals with TRD. Moreover, it is critical to elucidate a part of the molecular basis related to neurophysiology and neuroimaging findings to better understand the pathophysiology of TRD [24–26]. As such, we sought to explore the relationship between glutamatergic neural dysfunction and its associated gene expression profiles at the cellular level using a virtual histology approach. Our objectives were twofold: (1) to compare glutamatergic neurophysiological activity indexed by ICF TMS-EEG in the left DLPFC between participants with TRD and HCs and (2) to investigate the correlation between the cell-specific gene expression levels and difference of glutamate NMDA receptor-mediated signal propagation from the left DLPFC to the entire brain in TRD in comparison with HCs with the virtual histology approach. In this study, we hypothesized that glutamate NMDA receptor-mediated function indexed by ICF in the left DLPFC would be decreased in participants with TRD compared with HCs and that the disrupted glutamatergic neural propagation from the left DLPFC to the entire brain would correlate with gene expression level of every cortical cell type involved in glutamatergic regulation.

## Materials and methods

### Overview

**Fig. 1:**
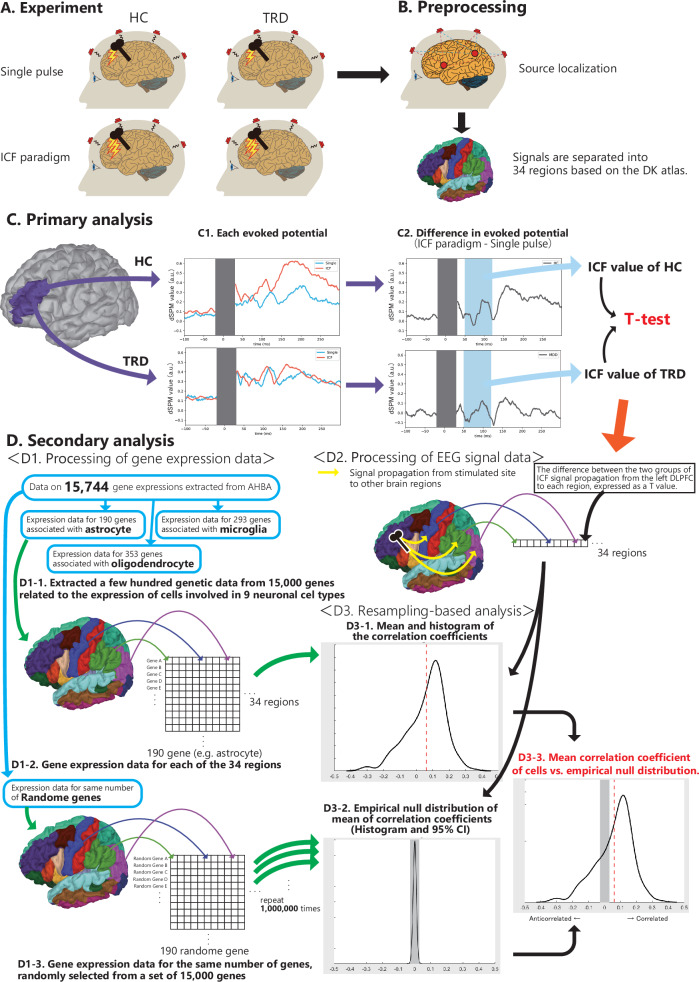
Overview of this research methodology. **A** All participants were examined with the ICF paradigm using the TMS-EEG method at the left DLPFC. **B** TEP were preprocessed and estimated signal source. Time series of current source density were calculated at the 34 regions of the brain based on the Desikan–Killiany atlas. **C** Panel C1 displays the time series of EEG current source density induced by the ICF paradigm and single-pulse TMS at the left DLPFC directly beneath the TMS stimulation. Panel C2 shows the difference between them, referred to as the ICF-dSPM current density. A *t*-test was applied to compare the ICF-dSPM current density within the time of interest (the blue bars) between the two groups. **D** Panel D1 outlines the processing of gene expression data in the AHBA dataset, consisting of 15,744 genes, mapped onto the 34 regions of the Desikan–Killiany atlas. The gene expression data classified into nine cell types by Zeisel et al was obtained (D1-1). To represent glutamatergic signal propagation from the left DLPFC to the other regions, the intergroup difference in ICF-dSPM current density at the time of interest was calculated as a *t*-value in all 34 regions (D2). A resampling-based approach was used to evaluate the statistical correlations between gene expression data and differential glutamatergic signal propagation for each cell. Panel D3-1 represents the mean and histogram of the correlation coefficients for a particular cell. The same number of genes as the target cells were randomly selected from the pool of 15,744 genes, with the mean correlation coefficient calculated in the same manner (D1-2). This process was repeated to generate an empirical null distribution of the mean correlation coefficients (D3-2). If the mean value of the correlation coefficient for a cell exceeds the 95% CI of the empirical null distribution, it is considered that the correlation between gene expression and differential glutamatergic signal propagation in that cell is significant. AHBA Allen human brain atlas, DLPFC dorsolateral prefrontal cortex, dSPM dynamic statistical parametric maps, EEG electroencephalography, HC healthy control, ICF intracortical facilitation, TEP TMS-evoked potential, TMS transcranial magnetic stimulation, TRD treatment-resistant depression.

The schematic representation of this investigatory approach is depicted in Fig. 1.

### Participants

This cross-sectional study was conducted at Keio University Hospital from 2017 to 2022. All participants provided written informed consent in accordance with the Declaration of Helsinki, and the study protocol (UMIN000028863) was approved by the Ethics Committee of the Keio University School of Medicine. Participants were eligible to participate if they met the following inclusion criteria: (1) a diagnosis of MDD as defined by the Diagnostic and Statistical Manual of Mental Disorders, Fifth Edition [27]; (2) aged 18 years or older; (3) receiving regular clinical treatment at Keio University Hospital, (4) a history of treatment failure with at least two previous antidepressants, as determined by a score of 3 or higher on the antidepressant treatment history form [28]; and (5) current severity of depression as indicated by a score of 18 or higher on the Montgomery Åsberg Depression Rating Scale (MADRS) [27–29]. Participants were excluded if they had: (1) a history of substance use disorders within the previous 6 months; (2) any contraindication for magnetic resonance imaging (MRI) or TMS; (3) an unstable physical illness or neurological condition; (4) a history of convulsive seizures or epilepsy; or (5) cognitive impairment as assessed by the mini-mental state examination [30]. Due to the concurrent participation of all subjects in another clinical trial (jRCTs032180188), which necessitated adjustments in medication regimens, the antidepressant administered in this study was standardized to venlafaxine at a dose range of 150–225 mg/day, while other antidepressant medications were tapered off or discontinued. The subjects underwent a four-week lead-in period prior to inclusion.

The screening of HCs was carried out by three certified psychiatrists (YN, SN, and MW) to confirm the absence of a history of psychiatric disorders through the administration of the structured clinical interview for DSM disorders, which served as the inclusion criterion for this cohort [31].

The participants in each group were matched for age and sex. The sample size calculation was based on the result of a previous study comparing TMS-EEG indices between individuals with MDD and HCs, with a delta of 37.53, standard deviation of 61.69, alpha of 0.05, and a desired power of 80% [32]. The proportion between the TRD and HC groups was adjusted to be 2:1 to ensure adequate recruitment. It was determined that 60 participants in the TRD group and 30 participants were necessary for accurate analysis.

### Clinico-demographic assessments

The medical history, years of education, and other relevant clinical data were obtained through the administration of structured interviews. The severity of depression was then evaluated by trained psychiatrists and clinical psychologists utilizing the MADRS.

### MRI data acquisition

All participants underwent MRI scans using a 3-T Siemens Prisma scanner with a 32-channel head coil. The scan was performed using T1-weighted magnetization-prepared rapid acquisition with gradient echo images, with the following parameters: echo time of 2.08 ms, repetition time of 1620 ms, inversion time of 1000 ms, flip angle of 8°, field of view of 232 mm, a matrix size of 186 × 192, and slice thickness of 1.25 mm.

### TMS administration

A 70 mm diameter figure-of-8 butterfly coil (DuoMAG 70BF; DEYMED Diagnostic Ltd.) was utilized for TMS with a monophasic TMS stimulator (the DuoMAG MP stimulator: DEYMED Diagnostic Ltd., Hronov, Czech Republic). High-resolution T1-weighted images were imported into the Brainsight TMS Navigation system (Rogue Research Inc.) and registered to digitized anatomical landmarks for online monitoring and coil localization. The resting motor threshold (RMT) was determined by recording a surface electromyogram from the first dorsal interosseous muscle of the right hand and identifying the optimal stimulation site over the left primary motor cortex. This threshold was defined as the minimum intensity required to elicit a motor-evoked potential of 50 μV or greater in the target muscle on at least 50% of all trials to the left primary motor cortex while wearing an EEG cap.

Single-pulse and paired-pulse TMS (interstimulus interval of 10 ms or ICF paradigm) were delivered to the left DLPFC of each participant with 5 s (±0.5 s) intervals, in accordance with established methods [33] (Fig. 1A). Specifically, 80 single-pulse and paired-pulse TMS were randomly delivered to each participant at an intensity of 120% of the RMT for the test pulse and 80% of the RMT for conditioned pulse. The coil was positioned at a 45-degree angle to the midline during TMS stimulation and the stimulation site was identified individually using an MRI-guided neuronal navigation system (Brainsight, Rogue Research Inc. Montréal, QC, Canada), located at Montreal Neurological Institute coordinates of [*x* = −38, *y* = 44, *z* = 26]. The coordinates were identified by a previous study based on its strong anticorrelation with the subgenual cingulate, which is thought to be associated with the pathophysiology of depression, and is expected to provide better therapeutic effects than TMS treatment of the stimulation site using the classic 5-cm rule [34]. To suppress auditory evoked potentials induced by TMS click sounds, a white noise masking method was applied to all participants using an earplug-type sound stimulator, with volume adjusted individually to cancel out the TMS clicking sound during stimulation [35].

### EEG recording and preprocessing

EEG was recorded using a TMS-compatible 64-channel amplifier with a sample-and-hold circuit system (TruScan LT, DEYMED Diagnostic s.r.o., Hronov, Czech Republic). We also used an EEG cap equipped with silver C-ring slit electrodes (the TruScan Research EEG Caps, 64-channel, DEYMED Diagnostic s.r.o., Hronov, Czech Republic). The electrodes were referenced to the right earlobe and the ground electrode was placed on the left earlobe. To ensure optimal signal quality, the impedance between the scalp and electrodes was maintained at less than 5 kΩ during the experiment. The sampling rate of each scan was 3 kHz.

TMS-EEG data was processed utilizing EEGLAB v2021.0 and TMS-EEG Signal Analyzer (TESA v1.1.1) [36, 37] and customized scripts executed on MATLAB software (R2020a, the MathWorks Inc., Natick, MA, USA). EEG data was initially epoched between −2000 ms and 2000 ms. Subsequently, the average signal amplitude between −500 ms and −150 ms was subtracted as a baseline correction, following which electrodes with high variability, as determined by median *z*-scores exceeding 3, were automatically removed. In addition, epochs with excessive noise exceeding 1000 µV in amplitude were automatically eliminated, and the remaining noisy epochs were scrutinized and eliminated manually. The electrodes (F5, F3, F1, F7, AF3, FC3, and FC5) corresponding to the DLPFC stimulation site were pre-specified to not be subjected to automatic exclusion. EEG data from −5 ms to 30 ms was removed to avoid TMS pulse artifacts. One caveat here is that if the EEG data is left cut off in preprocessing, ringing artifacts will occur when downsampling and filtering are performed. Therefore, cubic interpolation was performed, followed by downsampling and filtering. The data was down-sampled to 1 kHz and underwent the first round of fast independent component analysis to identify and eliminate the physical TMS decay components. Subsequently, the data was filtered using a bandpass (0.5–100 Hz) and notch (48–52 Hz) filter. The removed channels were interpolated using spherical interpolation. To remove other artifacts such as eye blinks, eye movements, and muscle artifacts, a second round of independent component analysis (EEGLAB infomax (runica)) was applied, and finally, data was re-referenced to the overall electrodes.

### EEG analysis

EEG analysis was performed using minimum norm estimate (MNE) software [38]. The sensor-based analysis involved the calculation of the TMS-evoked potential (TEP) as the average of all trials at the left DLPFC site, obtained from the average of the F3, F5, and AF3 electrode sites. The ICF-TEP was then calculated as the difference between the TEP obtained from the ICF paradigm and the TEP obtained from the single pulse. Furthermore, the local mean field power of ICF (ICF-LMFP) was calculated as the difference in the square root of the square of the TEP between the ICF paradigm and a single pulse [39, 40].

The source-based analysis, which constituted the primary analysis, involved the TMS-evoked EEG source reconstruction performed using the MNE software [38] (Fig. 1B). The surface reconstructions were obtained with the aid of FreeSurfer v6.0.0 and a 3-layer boundary element method model. The source spaces were created with 4098 sources per hemisphere (http://surfer.nmr.mgh.harvard.edu/). The boundary element method surface and source space were then manually co-registered with the EEG sensor digitized in the Neuromag head coordinate frame, defined by the nasal bridge, and left and right anterior ear points. The signals generated by neural activity in the brain were calculated by applying the forward model and computing the inverse solutions with dynamic statistical parametric maps (dSPM) [41, 42]. The noise covariance was estimated from individual trials using the shrink covariance method with the time window prior to TMS as the baseline (−500 ms to −15 ms) [43]. The dSPM current density time series calculated by the EEG source reconstruction method was extracted from the left DLPFC site immediately below the TMS stimulus, based on the Desikan–Killiany atlas [44]. Finally, the difference between the dSPM current density time series obtained from the ICF paradigm and the single-pulsed time series was calculated as the ICF-dSPM current density time series (Fig. 1C).

### Statistical analysis for signal propagation analysis

The analysis was confined to a time interval of 50 ms and 120 ms, as determined by prior studies [23, 33]. The time window was predetermined based on previous research, which shows a significant peak around 100 ms after TMS [45]. The ICF indices, including ICF-TEP, ICF-LMFP, and ICF-dSPM current density, were calculated as the average over the aforementioned time window. Subsequently, *t*-tests were conducted to compare the indices between the two groups (Fig. 1C). The primary outcome of the present study was to determine the difference in the ICF-dSPM current density within the aforementioned time window between the two groups, in concurrence with the established hypothesis. The level of significance was established as *α* = 0.05. Additionally, correlation analyses between ICF-dSPM current density and clinical parameters including MADRS score, and duration of illness were also conducted in patients with TRD.

### Gene expression analysis and statistical analysis

The virtual histology approach was executed according to prior studies [24–26]. The rationale of this analysis is as follows: Given that brain regions with high baseline expression of disorder-linked genes, which are related to glutamatergic signal propagation, are more susceptible over the course of TRD, the ICF signal propagation from the left DLPFC to each region in TRD should decrease in the brain regions with high baseline expression of these genes compared with HC [46]. In other words, if the reduction of ICF signal propagation in patients with TRD compared with HC at each brain region is correlated with gene expression in each brain region in HC, the alteration of the gene set may contribute to the reduction of ICF signal propagation in patients with TRD. The flowchart for the analysis here is also summarized in Fig. 1D and Supplementary Fig. 1. To conduct cell-specific gene expression analysis, post-mortem brain data from six donors was obtained from the Allen Human Brain Atlas (AHBA) genetics dataset [47]. The average gene expression data from the six AHBA donors was mapped to 34 regions in the Desikan–Killiany atlas, which was the same atlas employed in the source-based analysis of this study. The analysis was carried out in accordance with a practical guide for estimating local gene expression levels in the cortex and our previous studies [24, 48]. The rationale for using the mesoscopic parcellation of the cerebral hemispheres in this analysis (i.e., 34 regions) stems from the fact that the distributed method was used to estimate the signal sources in this study. Although the distributed method reduces spatial resolution, it is suitable for estimating activity in each region of the whole brain. Initially, gene assignment to the AHBA probe set using a re-annotator resulted in a probe set corresponding to 20,250 unique genes (1). For each brain, brainstem, and cerebellum samples were removed, and probes that did not exceed background in at least 50% of samples were excluded. Then, probes with the most consistent pattern of regional variation in the six donor brains were selected after quantification using differential stability measures (2,3). Using the same parcellation scheme applied to the cortical thickness data, samples were assigned to the cortical regions with a maximum distance threshold of 2 mm (4,5). Because the AHBA dataset had only two donors with right hemisphere data, only the left hemisphere was used for parcellation in our analysis. To reduce the donor-specific variation and focus on brain-related genes, a genetic filter based on stability differences was applied (2). As a result, the regional expression of 15,744 genes could be measured. The code utilized for this pipeline is available at: https://github.com/BM HLab/AHBAprocessing. Subsequently, the genes were classified into one of nine cell types, including ependymal cells, oligodendrocytes, microglial cells, CA1 pyramidal neurons, interneurons, endothelial cells, S1 pyramidal neurons, astrocytes, mural cells expressed in the cortex, based on data from Zeisel et al. utilizing single-cell RNAs from the somatosensory cortex (S1) and Cornu Ammonis 1 (CA1) region of the hippocampus in mice [49] (Fig. 1D1-1).

To correlate cell-specific gene expression profiles with differences in ICF between the two groups, the difference in ICF-dSPM current density between the two groups was analyzed using *t*-value indices for each of the 34 regions, which represent the differential glutamatergic neural propagation from the left DLPFC to the corresponding local region. First, the source reconstruction was applied to the 34 regions of the Desikan–Killiany atlas as described in the “Source-based EEG analysis” section, to obtain the ICF-dSPM current density in 34 brain regions. The differences in ICF-dSPM current density between the two groups were calculated using *t*-tests (“ICF-dSPM of HC group” vs. “ICF-dSPM of TRD group” within every 34 regions) (Fig. 1D2). Time of interest was defined between 50 ms and 120 ms. All 34 regions were included in the analysis because all regions are minimally connected to all other regions if weak connectivity is considered, and differences in excess or deficiency are crucial in this analysis.

Up until this point, the calculation of cell-specific gene expression profiles and differences in ICF-dSPM within each region had been performed. A resampling-based approach, based on previous studies, was subsequently employed to analyze the correlation between interregional profiles of cell-specific gene expression and altered ICF-dSPM within each region [25, 26].

The premise behind this was that if a particular gene set contributes to the differences in ICF signal propagation from the left DLPFC to each region between patients with TRD and HC, then the average correlation coefficient between regions between gene expression and ICF-dSPM alterations in that gene set should be significantly dissimilar from the average correlation coefficient obtained from a random gene-set (Fig. 1D3).

The mean correlation between the expression of genes involved in glutamatergic neurotransmission and the altered ICF-dSPM was calculated for 34 regions (Fig. 1D3-1). Significance was then evaluated through the utilization of an empirical null distribution of correlation coefficients between random genes and ICF-dSPM in the 34 regions (Fig. 1D3-2). This was achieved by repeating the process one million times. Subsequently, the proportion of average correlation coefficients that exceeded the correlation coefficients within null distribution correlation coefficients was calculated to obtain a two-sided *p*-value (Fig. 1D3-3). Given that this analysis was performed for each of the nine cells, the significance level was established at 0.0056 (0.05/9), as per the Bonferroni correction.

### Data sharing

All data requests should be submitted to the corresponding author for consideration. Access to anonymized data for scientific research may be granted following review.

## Results

### Demographic data

A total of 90 participants were enrolled in the study, comprising 60 participants with TRD and 30 HCs. There were no significant differences in age or sex between the two groups, as indicated by the demographic data presented in Table 1. However, one participant in each group was excluded from the analysis due to the presence of missing and incomplete data.

**Table 1:** Demographic data.

	TRD	HCs	statistics
Number of participants	60	30	–
Age (year old)	45.37 (11.85)	45.63 (13.16)	*t*_88_ = 0.096, *P*_uncorrected_ = 0.92
Sex, female (%)	40	40	*χ*^2^_88_ = 0.0, *P*_uncorrected_ = 1.0
Year of education (years)	15.3 (1.9)	14.7 (2.1)	*t*_88_ = 1.50, *P*_uncorrected_ = 0.14
MMSE score	29.1 (1.4)	28.5 (3.3)	*t*_88_ = 1.38, *P*_uncorrected_ = 0.17
Age of onset (year old)	36.0 (15.6)	–	–
Duration of illness (years)	10.9 (9.2)	–	–
MADRS score	32.1 (7.1)	–	–

Of note, the numbers in the table are shown as mean (±standard deviation). Others are shown as percentages.

*HCs* healthy controls, *MADRS* Montgomery Åsberg depression rating scale, *MMSE* mini-mental state examination, *TRD* treatment-resistant depression.

### Sensor-based analysis

ICF paradigm applied to the left DLPFC resulted in characteristic butterfly TEP plots in both the TRD and HC groups (Supplementary Fig. 2). No significant differences were observed in ICF-TEP or ICF-LMFP between 50 ms and 120 ms from the left DLPFC between the two groups (Supplementary Figs. 3 and 4) (TEP: *t*_86_ = 0.14, *p* = 0.88, Cohen’s *d* = 0.03; LMFP: *t*_86_ = −1.45, *p* = 0.15, Cohen’s *d* = 0.33).

### Source-based current density time series analysis

**Fig. 2:**
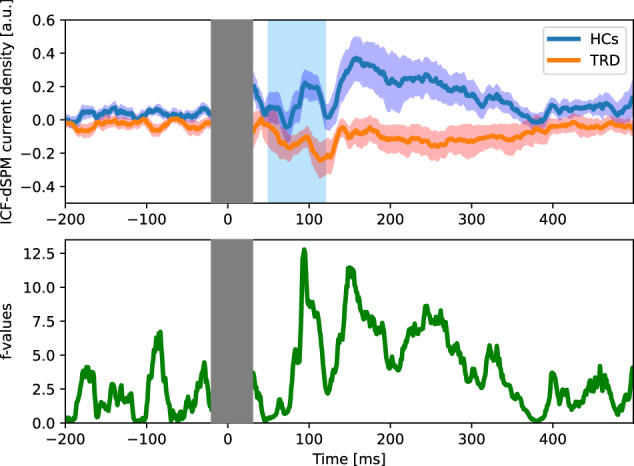
The waveform of ICF-dSPM current density time series at the left DLPFC. An orange and turkey blue line depict the waveform of the participants with TRD, and HCs, respectively. The shaded regions represent the variability in each stimulation condition as the standard error (upper panel). The *F*-value calculated with an analysis of variance, reflecting the difference in power between the two groups, is displayed in the lower panel. The post-stimulus interval of −15 ms to 30 ms is depicted as a gray bar due to data truncation, whereas the post-stimulus range of 50–120 ms, which is of interest, is highlighted by a blue bar. DLPFC dorsolateral prefrontal cortex, dSPM dynamic statistical parametric maps, HCs healthy controls, ICF intracortical facilitation, TRD treatment-resistant depression.

**Fig. 3:**
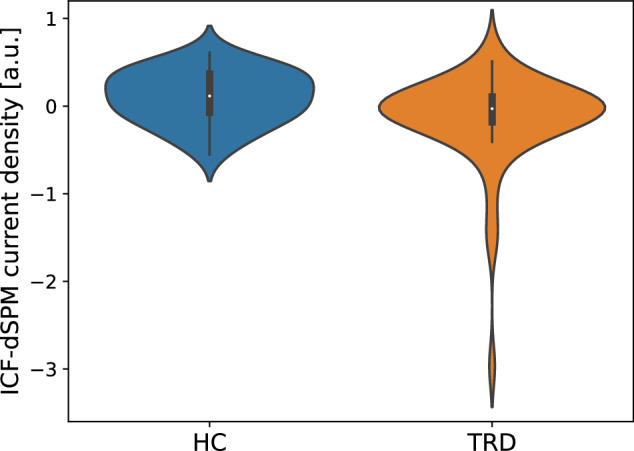
A violin plot of the ICF-dSPM current density at the left DLPFC. The results of the *t*-test comparison between the TRD and HC groups revealed a significant decrease in ICF-dSPM in participants with TRD (*t*_86_ = 2.27, *p* = 0.026, Cohen’s *d* = 0.52). DLPFC dorsolateral prefrontal cortex, dSPM dynamic statistical parametric maps, HC healthy control, ICF intracortical facilitation, TRD treatment-resistant depression.

The findings of the ICF-dSPM current density time series at the left DLPFC site directly beneath the TMS stimulation are shown in Figs. 2 and 3. ICF-dSPM current density between 50 ms and 120 ms was found to be lower in participants with TRD compared with HCs (*t*_86_ = 2.27, *p* = 0.026, Cohen’s *d* = 0.52). The correlation between ICF-dSPM current density and clinical parameters including MADRS score and duration of illness, was not significant (MADRS score: correlation coefficient = −0.090, *p* = 0.50; duration of illness: correlation coefficient = 0.045, *p* = 0.73).

### Gene expression analysis

**Fig. 4:**
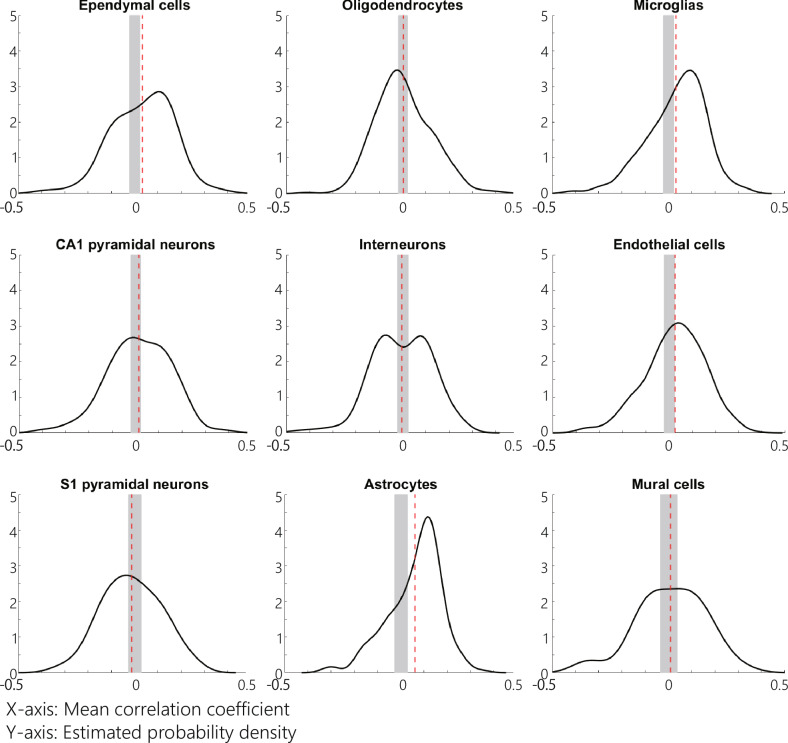
The mean correlation coefficients between the gene expression degree and the altered ICF-dSPM current density in the inter-regional profiles. The probabilistic disparities between the correlated probability distributions of the interregional profiles between gene expression levels in cell types primarily expressed in the nervous system and the altered signal propagation profiles of our data and the empirical null distribution (the interregional profile between random gene expression levels and our altered signal propagation profile). The *x*-axis signifies the mean correlation coefficients, while the *y*-axis symbolizes the estimated probability density of the mean correlation coefficients. The black line represents the estimated probability density function for the correlation coefficients between each cell type gene expression level and the signal propagation profiles of our data. The significance of the lower and upper cutoff are denoted by the vertical edges of the shaded gray box, while the mean correlation coefficients for each cell type are indicated by the dashed red lines. It was determined that after correcting for multiple comparisons, the following cell types significantly differed from the empirical null distributions: astrocyte (*p*_uncorrected_ < 0.0001; *α* = 0.0056), ependymal cells (*p*_uncorrected_ = 0.001; α = 0.0056), and microglial (*p*_uncorrected_ = 0.001; *α* = 0.0056). CA1 Cornu ammonis 1, dSPM dynamic statistical parametric maps, ICF intracortical facilitation, TMS transcranial magnetic stimulation, S1 somatosensory cortex.

The correlations between gene expression levels of specific cell types and altered ICF-dSPM current density across different regions are presented in Fig. 4. As depicted in Fig. 4, the mean correlation coefficients for astrocyte (*p*_uncorrected_ < 0.0001; *α* = 0.0056, average correlation coefficients = 0.062, CI = [−0.027, 0.030]), ependymal cells (*p*_uncorrected_ = 0.001; *α* = 0.0056, average correlation coefficients = 0.033, CI = [−0.022, 0.024]), and microglias (*p*_uncorrected_ = 0.001; *α* = 0.0056, average correlation coefficients = 0.034, CI = [−0.022, 0.024]) were found to be significantly different from the empirical null distributions, while no significant difference was observed in the other cell types after correcting for multiple comparisons (oligodendrocyte cells, *p*_uncorrected_ = 0.775; CA1 pyramidal neurons, *p*_uncorrected_ = 0.084; interneurons, *p*_uncorrected_ = 0.59; endothelial cells, *p*_uncorrected_ = 0.0064; S1 pyramidal neurons, *p*_uncorrected_ = 0.16; and mural cells, *p*_uncorrected_ = 0.55; *α* = 0.0056).

## Discussion

**Fig. 5:**
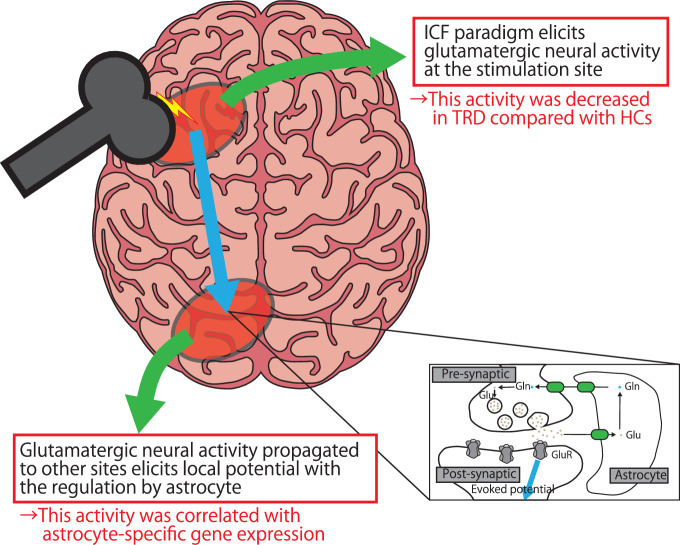
The overview and main results of this study. The application of the ICF paradigm to the left DLPFC elicits glutamatergic activity at the site of stimulation, and it is significantly decreased in participants with TRD compared with HCs. Furthermore, this glutamatergic activity is capable of propagating via axonal projections from the left DLPFC to other areas of the brain, indirectly releasing glutamate at synapses and potentially stimulating postsynaptic neurons. As the regulation of glutamate release and clearance at the synaptic level is primarily governed by astrocytes, the evoked potentials at each site of propagation could be influenced by the glutamate recycling capacity of astrocytes. In accordance with this hypothesis, our findings demonstrate that the decreased glutamatergic propagation from the left DLPFC throughout the brain may be related to a reduction in the expression of astrocyte-associated genes throughout the brain. This suggests that the glutamatergic propagation from the left DLPFC may be contingent upon astrocyte function in the brain. DLPFC dorsolateral prefrontal cortex, Gln glutamine, GluR glutamate receptor, HCs healthy controls, ICF intracortical facilitation, TRD treatment-resistant depression.

In the present study, we investigated the glutamatergic neural activity indexed by the ICF paradigm in the left DLPFC of participants with TRD compared with HCs. Furthermore, we explored the correlations between cell-specific gene expression levels and the alternation of ICF-dSPM in participants with TRD by utilizing the AHBA dataset. The result indicated that the neurophysiological function of the left DLPFC, primarily mediated by the glutamatergic NMDA receptor, was reduced in participants with TRD compared with HCs, with a medium effect size. Additionally, our findings indicate that disrupted glutamatergic signal propagation from the left DLPFC to the entire brain may be linked to a decreased level of gene expression of astrocyte-associated genes (Fig. 5).

The strengths of this study are as follows: (1) this is a pioneering study using the TMS-EEG method to reveal neurophysiological dysfunction mediated by glutamate NMDA receptors in participants with TRD; (2) the sample size of this study is relatively large for a TMS-EEG study of TRD; (3) the present study combined both high-density EEG electrodes and MRI to digitize both head and brain spatial information to improve the accuracy of TEP signal source estimation, thereby uncovering TEP findings that could not be revealed by sensor-level analysis by adding signal source-level analysis; and (4) glutamatergic neurophysiology in TRD, using TMS-EEG, as well as MRI and virtual histology approaches, provided insight into the possible contribution of reduced astrocyte expression levels to the lower ICF-dSPM in participants with TRD.

Interventional studies, such as ketamine and rTMS treatment, have demonstrated correlations between their therapeutic effects on TRD and glutamatergic neural function. A study using ^1^H-MRS indicated that ketamine promptly elevated glutamate levels in the anterior cingulate cortex in comparison to a placebo, in individuals with TRD [50]. Godfrey et al. applied rTMS to the left DLPFC in individuals with TRD and showed increased levels of glutamatergic neurometabolite as measured by ^1^H-MRS [51]. Additionally, a genome-wide association study revealed that the response to ketamine treatment was linked to single nucleotide polymorphisms related to glutamatergic function [52]. These findings, in conjunction with our results, suggest that the pathophysiology of TRD entails reduced glutamatergic NMDA receptor-mediated neural activities in the left DLPFC. Of note, our finding was inconsistent with previous ICF studies in MDD using TMS-EMG neurophysiology, which showed increased ICF in M1 of patients with MDD compared with HC [21]. This may be due to the difference in modalities between TMS-EEG findings for the DLPFC and TMS-EMG for M1 and the difference in pathophysiology between TRD and MDD.

ICF paradigm elicits excitation of the TEP at the stimulation site through the glutamate NMDA receptor-mediated activity [53]. Furthermore, the glutamatergic activities at the site of stimulation are propagated to other brain regions via axons extending from neurons in the left DLPFC, which release glutamate at synapses and activate postsynaptic neurons at each brain region. Hence, the TEP in each region throughout the brain represents the glutamatergic neural propagation from the left DLPFC to the corresponding local region. In addition, our study found a correlation between the astrocyte expression level in HC and the relative reduction in glutamatergic signal propagation in TRD compared with HCs. It has been noted that astrocytes play a substantial role in the regulation of glutamate release and clearance at the synaptic level, although the association between gene expression and ICF-dSPM was not sufficiently guaranteed, the diminished glutamatergic propagation from the left DLPFC to local brain regions driven by ICF paradigm may result from decreased levels of astrocyte expression in TRD [54] (Fig. 5). In fact, astrocytes dysfunction has been implicated in the etiology of depression [55]. It is hypothesized that the brain areas with higher levels of expression of disease-related genes at baseline are more prone to develop disease progression [46, 55]. Thus, reduced levels of astrocyte-related gene expression across these brain areas could potentially lead to diminished signal propagation of glutamatergic neurophysiological functioning, a characteristic observed in TRD. However, it is important to note that this is merely a correlation. While there is a possibility of a relationship between gene expression and ICF-dSPM, establishing a definitive causal link requires comprehensive large-scale studies encompassing genetics and neurophysiology within the same subjects.

In the present study, the sensor-level analysis showed no significant group differences, but the source-level TEP analysis revealed significant differences between the two groups. In TMS-EEG measurements, white noise masking can reduce the influence of auditory stimuli from the TMS coil. However, residual noise, including TMS-induced muscle contractions, is difficult to eliminate completely because it can spread throughout the brain by volume conduction. In addition to the impacts of contamination, it is noteworthy that extensive or tangential neural activity can also transmit signals to distant electrodes in a similar manner. Therefore, source-level analysis can reduce the effect of volume conduction as much as possible, which may improve the accuracy of the analysis of the target brain region.

Several limitations are inherent in the present study. Firstly, the simultaneous induction of peripheral nerve stimulation-derived brain activity on TEP raises the concern that not all TEP findings necessarily reflect cortical stimulus-derived neural firing activity alone. Secondly, the observed difference in TEP between the TRD and HC groups may, to some extent, be attributed to the effects of the antidepressant. Thirdly, the examination of the left DLPFC only, as a result of the experimental design, precludes the examination of a comprehensive view of the pathophysiology of TRD. A more exhaustive analysis incorporating TMS over not only the left DLPFC but also the other brain regions as regions of interest would significantly enhance the understanding of the pathophysiology of TRD. Fourthly, in the present study, a navigation system based on individual T1 MRI images was used for signal source estimation, but the location of individual electrode sites was not registered in the navigation system. Instead, we provisionally determined the position of each electrode site based on the 10–20 international system. However, this approach may reduce the accuracy of signal source estimation. Fifth, the time windows to measure signal propagation were set between 50–120 ms, equal to the time window used to measure the response at the stimulated area. Since the response due to signal propagation should be delayed by a few ms compared to the response at the stimulated site, the time window might have been set correspondingly later by that amount. However, it is known that auditory and somatosensory evoked potentials start around 130 ms and peak around 200 ms regardless of the brain region, so we could not delay the time windows [56]. Sixth, ICF-dSPM current density comes from evoked potentials elicited by TMS. Therefore, it includes not only direct signal propagation but also indirect propagation. Seventh, the accuracy of source localization could be a potential limitation of the analysis. The inverse problem, which involves reconstructing the sources from scalp EEG signals, is ill-posed and can include ambiguities. Additionally, source localization is sensitive to the accuracy of electrode placement, which was not individualized but based on a template in our analysis. These factors can affect the reliability and interpretability of the estimated sources and may lead to biased or distorted conclusions. Finally, since the genetic information obtained from the AHBA postmortem brain database is not from the participants in this study, the finding from the AHBA database analysis should be considered only as a reference finding in explaining the molecular basis behind the TMS-EEG.

In conclusion, the present study uncovered that glutamatergic neurophysiological function, as indexed by the ICF paradigm in the left DLPFC, was diminished in participants with TRD compared with HCs. Moreover, the virtual histology investigation suggests that impaired glutamate NMDA receptor-mediated neural propagation from the left DLPFC to the entire brain might be attributable to a decreased expression level of the astrocyte-related gene in this population. These findings suggest that impaired glutamatergic neurotransmission may be a biological hallmark of TRD, which may be essential to developing diagnostic aids and therapeutic strategies rather than only operational diagnostic criteria by clinical symptoms.

## Supplementary information

Supplementary Figures

## Data Availability

The data presented in this study are available upon reasonable request from the corresponding author (YN).
