# Supplementary material for: Decreased prefrontal glutamatergic function is associated with a reduced astrocyte-related gene expression in treatment-resistant depression
Source: Transl Psychiatry. 2024 Nov 25;14:478. doi: 10.1038/s41398-024-03186-2 (PMC11589749; doi:10.1038/s41398-024-03186-2)
Supplement: Supplementary file 1 — Supplementary Figures [file 41398_2024_3186_MOESM1_ESM.docx]

**Supplementary Figures**


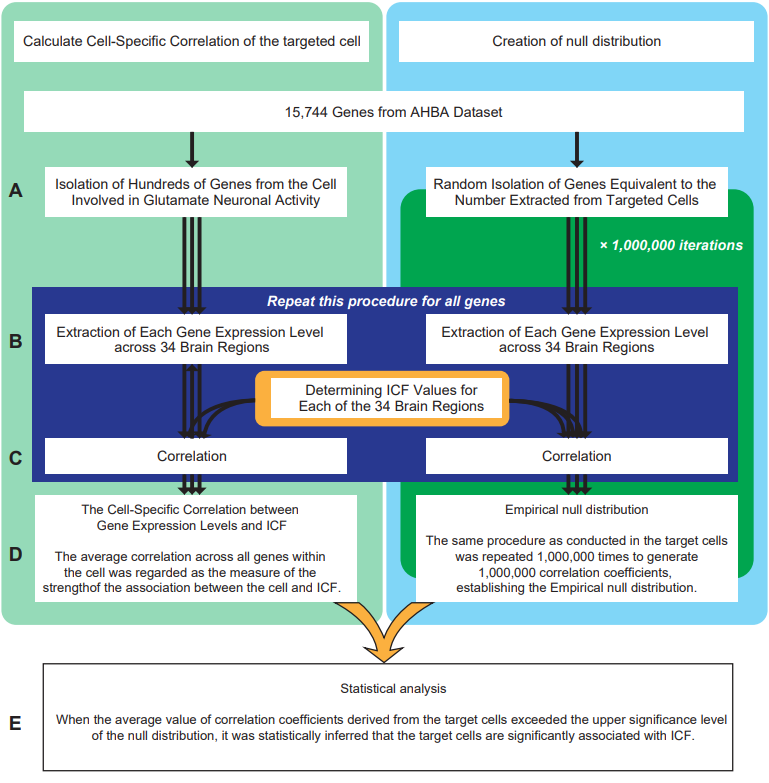


**Supplementary Figure 1**. **Overview of virtual histology approach.** This figure describes the procedure of our virtual histology approach. The left column illustrates the process for a specific target cell, while the right column is for creating the empirical null distribution for statistical processing. (Left column) First, we extract the genes related to a specific cell from AHBA data (A). For each gene, we calculate the expression levels across 34 brain regions (B). We then compute the correlation between the distribution of these 34-region expression levels and the between-group difference in the ICF values derived from source estimation (C). This is done for all genes of that cell (BC). The average correlation coefficient for each gene is subsequently determined (D). (Right column) To create the empirical null distribution, we randomly extract the same number of genes instead of extracting genes specific to a certain cell. The subsequent steps are the same as those done for the target cell. This procedure is repeated 1,000,000 times. Therefore, from the target cell, we get one average correlation value, and from the empirical null distribution, we get 1,000,000 average correlation values. We then test how statistically strong the average correlation from the target cell is against the empirical null distribution (E).

AHBA: Allen Human Brain Atlas; ICF: intracortical facilitation


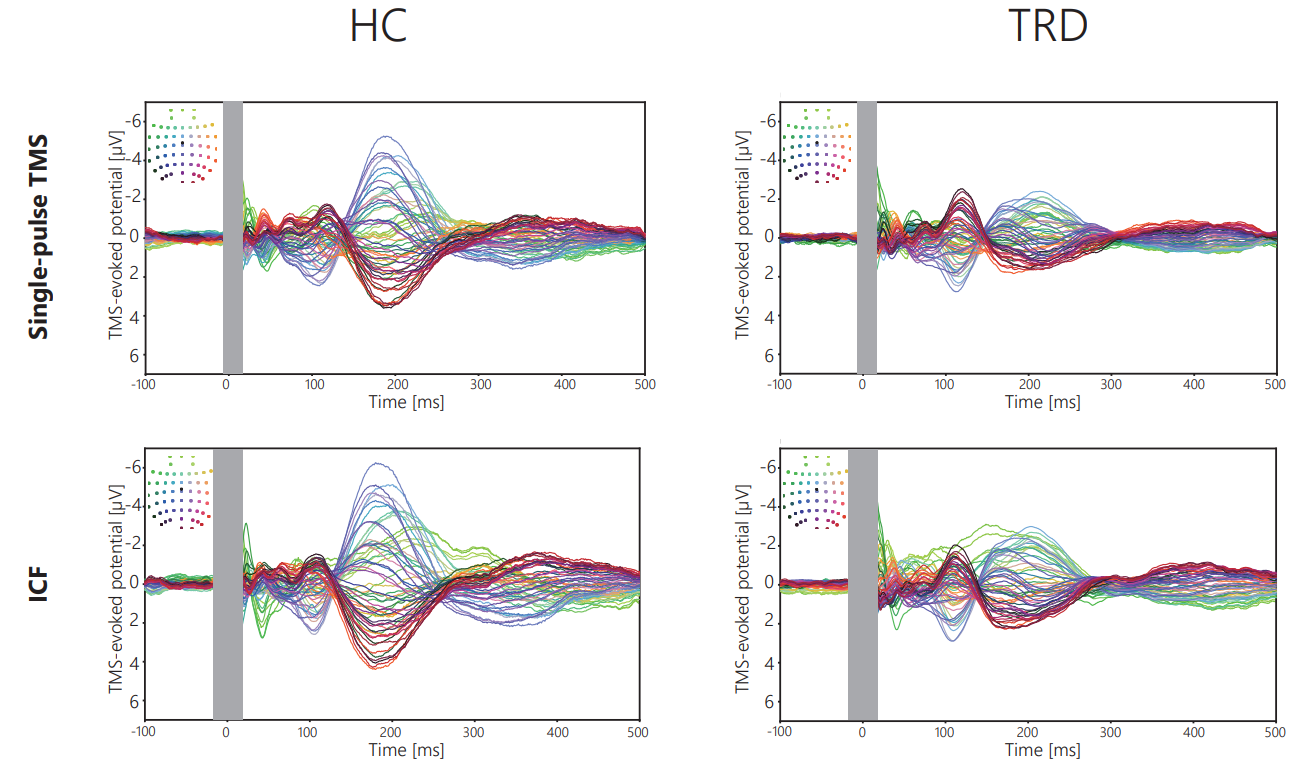


**Supplementary Figure 2. The butterfly plots of TEP waveforms.** Average single pulse (upper) and ICF paradigm (lower) conditions from all participants are presented for both HCs (left) and patients with TRD (right). The TMS post-stimulus interval, which ranges from -5 to 30 milliseconds for single pulse and -15 to 30 milliseconds for ICF paradigm, is depicted as a grey bar due to data truncation. The coloring of each waveform in the butterfly plots aligns with the electrode color diagram found in the upper left corner.

HCs: healthy controls; ICF: intracortical facilitation; TEP: TMS evoked potential; TMS: Transcranial magnetic stimulation; TRD: treatment-resistant depression.


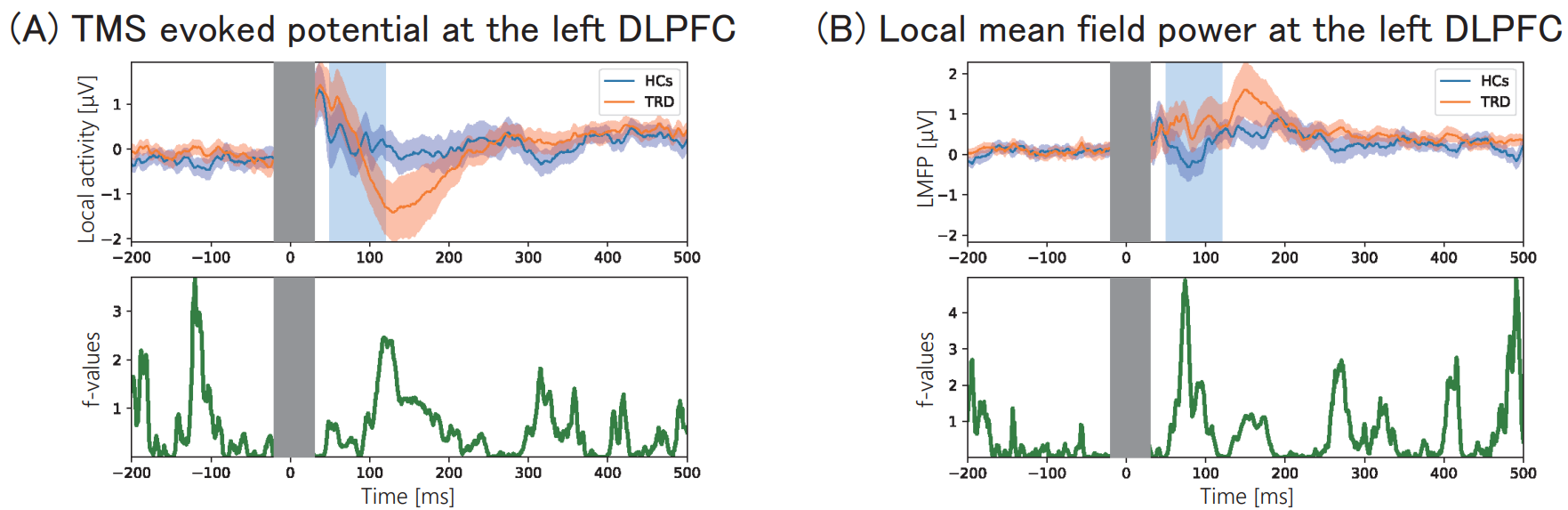


**Supplementary Figure 3**. **The waveform of TMS-evoked local activities.** The waveform of the TEP is displayed in Panel A, while that of the LMFP is shown in Panel B, with an orange line for participants with TRD and a turquoise blue line for HCs, both at the site of stimulation (i.e., DLPFC). The shaded areas in each group and panel depict the variability in each stimulation condition as the standard error (upper panel), while the lower panel represents the F-values from an analysis of variance for the power difference between the two groups. The gray bars in the figure indicate the data cutoff range from -15 ms to 30 ms before and after TMS. The blue bars represent the range from 50 to 120 ms post-stimulus, which is the time of interest for this analysis. HCs: healthy controls; LMFP: local mean field power; TEP: TMS-evoked potential; TMS: transcranial magnetic stimulation; TRD: treatment-resistant depression


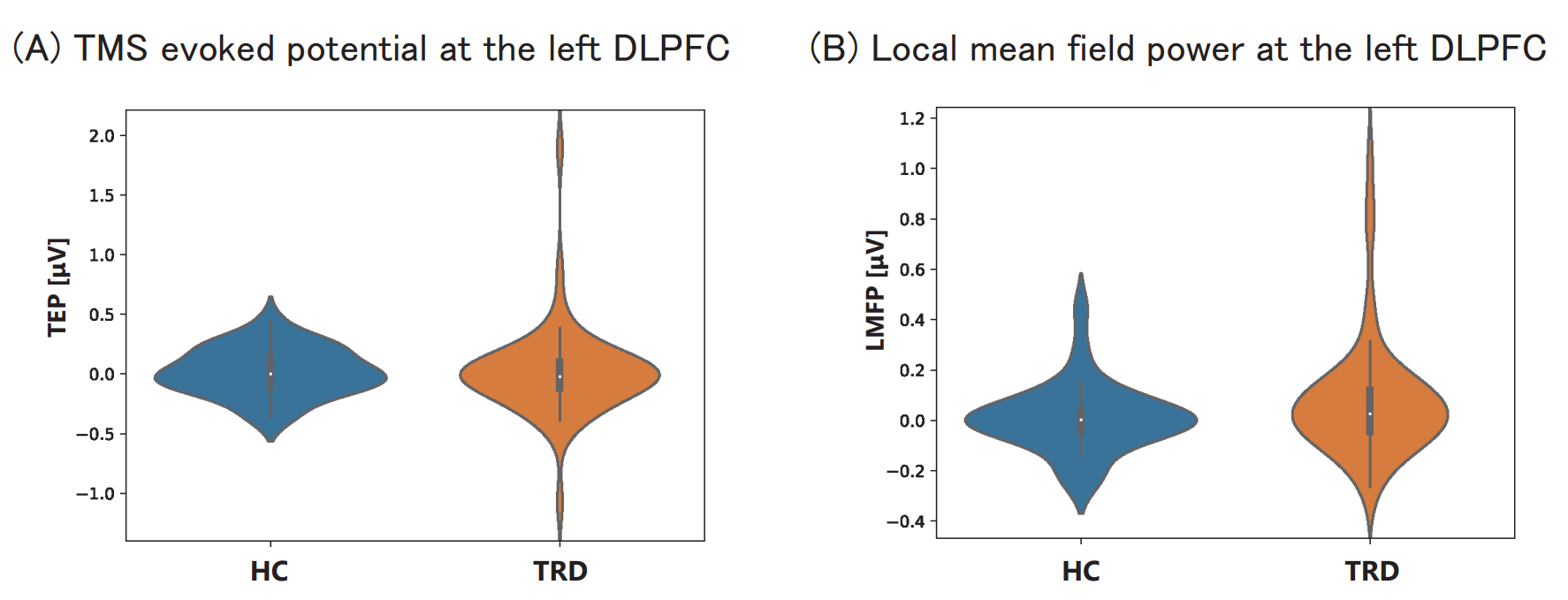


**Supplementary Figure 4**. **The violin plot of the ICF-TEP (left) or ICF-LMFP (right) at the left DLPFC.** The results of the t-test comparison between the TRD and HC groups indicate a lack of significant difference in either analysis, with TEP exhibiting t86 = 0.14, p = 0.88, and Cohen's d = 0.03; LMFP exhibiting t86 = -1.45, p = 0.15, and Cohen's d = 0.33.

DLPFC: dorsolateral prefrontal cortex; HC: healthy controls; ICF: intracortical facilitation; LMFP: local mean field power; TEP: TMS-evoked potential; TMS: transcranial magnetic stimulation; TRD: treatment-resistant depression
